# Resource Competition Between Melon Fly, *Zeugodacus cucurbitae* and Oriental Fruit Fly, *Bactrocera dorsalis*

**DOI:** 10.3390/insects17090921

**Published:** 2026-09-02

**Authors:** Hongai Su, Jin Zhao, Zhenyu Hao, Haikuo Yu, Saleem Jaffar, Guangwen Liang, Ling Zeng, Muhammad Asghar Hassan, Yongyue Lu

**Affiliations:** 1 Department of Entomology, South China Agricultural University, Guangzhou 510642, China; 2 School of Preclinical Medicine, Zunyi Medical University, Zunyi 563006, China; 3 State Key Laboratory of Biocontrol, School of Ecology, Sun Yat-Sen University, Shenzhen 518000, China

**Keywords:** *Zeugodacus cucurbitae*, *Bactrocera dorsalis*, survival rate, dry weight, competition, host plants

## Abstract

The melon fly, *Zeugodacus cucurbitae*, and the oriental fruit fly, *Bactrocera dorsalis*, are economically important tephritid pests whose sympatric distribution and overlapping host ranges precipitate intense interspecific competitive interactions. To explore the interspecific competition during larval development between these two species, we conducted a comparative study under controlled laboratory conditions. Larvae were evaluated on five host fruits—pumpkin (*Cucurbita moschata*), cucumber (*Cucumis sativus*), winter melon (*Benincasa hispida*), bitter gourd (*Momordica charantia*), and guava (*Psidium guajava*)—across six larval density gradients (4.0, 1.33, 0.8, 0.57, 0.44, and 0.36 larvae g^−1^ of host tissue). In mixed-species treatments, *Z. cucurbitae* exhibited significantly higher survival rates and greater adult dry weights than *B. dorsalis* on all cucurbit hosts except guava, indicating a consistent competitive advantage. Under high-density conditions, mixed-species treatments displayed stronger competitive effects than single-species treatments. Survival rate and adult dry weight of both species were significantly affected by the interactive effects of host plant species and larval density, with these factors jointly determining competitive outcomes. These findings improve our understanding of interspecific competition in tephritid fruit flies and provide a scientific basis for developing more effective management strategies for these economically important pests.

## Simple Summary

Melon fruit fly and oriental fruit fly are two major pests of cucurbit crops, inflicting substantial economic losses and impacting agricultural productivity and livelihoods. This study explored larval feeding competition between two fruit fly species on five common host plants: pumpkin, cucumber, winter melon, bitter gourd, and guava, under varying larval densities to understand the population dynamics. Results demonstrated that *Z. cucurbitae* exhibited a significant competitive advantage in relation to *B. dorsalis* in mixed groups under cucurbit host conditions and most density gradients. The study revealed that when the two fruit fly species coexisted, they exhibited notable differences in survival rates and adult dry weight compared to when they were alone. These variations were influenced by host plant species and larval density, offering valuable insights into the competitive dynamics between the species during the larval stage.

## 1. Introduction

Interspecific competition is a fundamental ecological process that profoundly shapes the population dynamics and ecological distribution of insect species, particularly when they share overlapping niche requirements [1]. The melon fruit fly, *Zeugodacus cucurbitae* (Coquillett, 1899), and the oriental fruit fly, *Bactrocera dorsalis* (Hendel, 1912), are two economically significant pests of cucurbit and fruit crops, which belong to the family Tephritidae, order Diptera [2,3]. These two fruit fly species can co-infest the same host plants and share similar feeding and reproductive ecologies [4,5,6]. Under suitable environmental conditions, the complete life cycle of both fruit fly species lasts approximately 30 to 90 days, with the larval stage persisting for 7–14 days. The mature wild-type adults typically initiate mating and oviposition approximately 10 days after emergence. Female adults deposit eggs into the fruit pulp using their ovipositors, and the subsequently hatched larvae feed on the pulp tissue [7,8]. The larval phase constitutes the most destructive developmental period, with third-instar larvae showing maximal feeding intensity that directly induces fruit tissue maceration and premature abscission [9,10]. Their pronounced niche overlap between these sympatric species facilitates multi-stage competitive interactions during co-infestation events, ranging from adult oviposition contests to larval resource competition. These interspecific interactions substantially alter key life history traits in both species and induce cascading effects on host plant physiological performance and crop yield potential [11,12,13].

Previous studies by Xingxing et al. [14] have offered valuable insights into interspecific competition among fruit fly larvae. It has been demonstrated that such competition can involve various factors, including food resources, living space, and chemical signal interference, all of which can significantly affect larval growth, development, and population dynamics. For instance, on pumpkin, when *Z. cucurbitae* and *Z. tau* coexist, *Z. cucurbitae* exhibits greater tolerance to crowding and gains a competitive advantage in interspecific competition. Similarly, in competition between *B. dorsalis* and *Bactrocera correcta*, *B. dorsalis* can gain a competitive edge by adjusting its life history strategies [15]. However, these studies have primarily focused on competition between *B. dorsalis* and other related species, leaving a significant knowledge gap concerning the specific competitive mechanisms between *Z. cucurbitae* and *B. dorsalis* larvae. For two economically important fruit fly species with highly overlapping niches, does larval competition on shared host plants have consequences across developmental stages on adult survival rates and dry weight? Addressing these questions is critical for elucidating the mechanisms underlying tephritid interspecific competition and developing science-based population management strategies.

Recent studies have demonstrated that intraspecific competition regulates insect adaptive responses through the interplay between nutritional environment and larval density. Specifically, Morimoto et al. [16] revealed that the interaction among uric acid metabolism, larval crowding, and dietary nutrition significantly influences the development and reproduction of *Drosophila melanogaster* Meigen, 1830 (Diptera: Drosophilidae). Similarly, Villafán et al. [17] demonstrated that polyphagous tephritid fruit flies exhibit nonlinear phenotypic responses under high larval density and extreme nutritional substrate conditions (e.g., varying protein + carbohydrate content and protein-to-carbohydrate ratios), indicating that food quality and density collectively represent insect fitness. These findings provide a theoretical basis for understanding the cross-developmental stage effects of larval competition between *Z. cucurbitae* and *B. dorsalis* on shared host plants in this study.

In this study, we aim to bridge this gap by conducting controlled experiments to investigate the interspecific competition between *Z. cucurbitae* and *B. dorsalis* larvae. By implementing six distinct density gradient experimental setups, we quantified both survival proportions and dry mass changes of larvae from the two fruit fly species across five common host plant species throughout their complete larval-to-adult developmental period, allowing for a comprehensive analysis of larval competition dynamics. These findings provide a scientific basis for understanding ecology, survival, and resource competition.

## 2. Materials and Methods

### 2.1. Colony Establishment and Maintenance

Larvae of *B. dorsalis* and *Z. cucurbitae* were collected from bitter melon fields at the teaching experiment site of South China Agricultural University (latitude 23.1598° N, longitude 113.3449° E). After collection, uniform 48 h post-hatching first-instar larvae were transferred individually via fine camel hair brushes onto the surface of homogenized fruit mesocarp substrate to avoid mechanical injury; larvae were evenly distributed across the substrate surface immediately after transfer. The larvae were first reared in plastic containers (22 × 15 × 7.9 cm) provisioned with fresh *Cucurbita pepo* slices as the host substrate. When larvae reached the late third instar (typically 5–6 days post-collection), they were individually transferred to pupation buckets (with a diameter of 34.5 and a height of 32 cm) containing sterilized fine-grained sand to complete pupal development under controlled conditions [18]. The larvae were kept at a temperature of 27 ± 1 °C, a relative humidity of 75 ± 1%, and a photoperiod of 14:10 (light–dark) in a climate-controlled chamber. After the adults emerged, they were provided with an artificial diet consisting of a 1:1 (*w*/*w*) mixture of yeast extract and dry sugar and housed in wooden cages measuring 35 × 35 × 35 cm [19,20]. Fresh slices of pumpkin (5 × 5 × 1 cm) were placed into the adult cages at the peak oviposition period (around 20 days) and then removed after 4 h, following Gaddanakeri et al. [7] and Huang et al. [8].

### 2.2. Experimental Setup

#### 2.2.1. Host Preparation

To investigate the competitive ability of larvae on hosts by establishing different larval density gradients through varying host weight gradients, fresh host fruits of uniform maturity were subjected to triple rinsing with distilled water, followed by removal of exocarp and seeds while retaining mesocarp (larva-preferred) tissue for experimental use. First-instar larvae used in the assays were obtained and hatched under controlled environmental conditions. Individually, larvae of uniform size were gently transferred onto the prepared host substrates using a fine camel-hair brush, after which they were co-incubated with quantitatively matched host substrates in 250 L polystyrene rearing chambers (pre-lined with sterile absorbent filter paper to retain putrefactive exudates) [5,21]. Based on preliminary results (using pumpkin as the test host, where host weight below 5 g caused larval developmental delays, while exceeding 55 g led to resource surplus), two experimental groups were established.

#### 2.2.2. Larval Allocation and Density Treatments

The number of larvae per gram was calculated in all experimental samples, and then they were placed in 10 cm^3^ plastic containers (pre-lined with a single layer of absorbent filter paper at the bottom) and reared under controlled conditions. Each treatment was replicated five times. Larvae of uniform size (48 h post-oviposition) from both species were collected for subsequent experiments. The experimental hosts included five species: pumpkin (*Cucurbita moschata*), cucumber (*Cucumis sativus*), winter melon (*Benincasa hispida*), bitter melon (*Momordica charantia*), and guava (*Psidium guajava*).

#### 2.2.3. Experimental Groups

Single group: Six host weight gradients (5, 15, 25, 35, 45, and 55 g) were used, corresponding to larval densities of 4.0, 1.33, 0.8, 0.57, 0.44, and 0.36 larvae/g, respectively (Table 1). Each container was stocked with 20 newly hatched larvae of either *B*. *dorsalis* or *Z*. *cucurbitae* to evaluate single growth and development patterns. Mixed group: Following the same density gradient design, six gradients (10, 30, 50, 70, 90, and 110 g) were established, with each container simultaneously introduced with 20 *B. dorsalis* and 20 *Z. cucurbitae* larvae to study interspecific interaction effects [21].

### 2.3. Analysis of Adult Survival Rate and Dry Weight

Larvae of two tephritid species were reared at different host densities until they reached the third-instar stage. They were then transferred to a pupation substrate consisting of moist sand (about 40% humidity) in a 25 L tub using a No. 10 mesh sieve and maintained at a constant temperature of 27 °C to complete pupation. The total number of surviving adults of each species was recorded in each replicate cage.

Mixed group: Each box contained 20 first-instar larvae of the oriental fruit fly and 20 first-instar larvae of the melon fruit fly. Six host-resource gradients and 5 biological replicates were established to assess interspecific interactions under shared host conditions.

Control group: Briefly, 20 larvae of the melon fruit fly and 20 larvae of the oriental fruit fly were individually reared in six host mass gradients of 5 g, 15 g, 25 g, 35 g, 45 g, and 55 g, with each group having 20 replicates per treatment, to evaluate the growth and development patterns of the species under varying host-resource availability.

Subsequently, adults 1–2 days post-emergence were dried to a constant weight in a 60 °C electro-thermostatic blast oven (OHG-914385-III, Shanghai CIMO Medical Instrument Manufacturing Co., Ltd., China). Individual dry weights were measured using an analytical balance (FA114, Shanghai Haikang Electronic Instrument Factory) with a precision of 0.0001 mg. Each treatment combination was biologically replicated six times to ensure statistical reliability.

### 2.4. Statistical Analyses

All values are presented as mean ± standard error of the mean (SEM), and statistical significance was set at *p* < 0.05. All statistical analyses were performed using DPS 9.5 (Data Processing System, China) and R version 4.3.2 (R Core Team, 2023).

Prior to statistical analysis, adult survival rate data (proportional data ranging from 0 to 1) were arcsine-square-root-transformed to meet the assumptions of normality and homogeneity of variance required for parametric tests. The transformation was performed using the formula transformed value = arcsine (√proportion). This transformation is specifically recommended for proportional data to stabilize variance and normalize the distribution, particularly when survival rates approach 0% or 100%. After transformation, the Shapiro–Wilk test confirmed that the data met normality assumptions (*p* > 0.05).

Two types of datasets were analyzed: (1) transformed adult survival rate data and (2) adult dry weight (continuous data). For both datasets, multifactorial ANOVA was performed with species (*Z. cucurbitae* vs. *B. dorsalis*), host plant (five levels), larval density (six levels), and group type (single vs. mixed) as fixed factors, with all two-way and three-way interaction terms initially included. Prior to analysis, normality (Shapiro–Wilk test) and homogeneity of variance (Levene’s test) were assessed to confirm parametric assumptions were met. For dry weight data, these assumptions were satisfied without transformation. Where significant interactions were detected, simple main effects were further analyzed at each factor level. Pairwise comparisons were conducted using Tukey’s HSD post hoc tests.

Because larvae of both sexes were reared within the same experimental unit, all statistical analyses treated each replicate container (rather than individual insects) as the experimental unit to avoid pseudo-replication.

Full ANOVA results, including degrees of freedom (df), F values, and *p* values, are provided in Table 2.

## 3. Results

### 3.1. Comparative Analysis of Adult Survival Rates in Mixed Groups Across Five Host Plants

**Figure 1:**
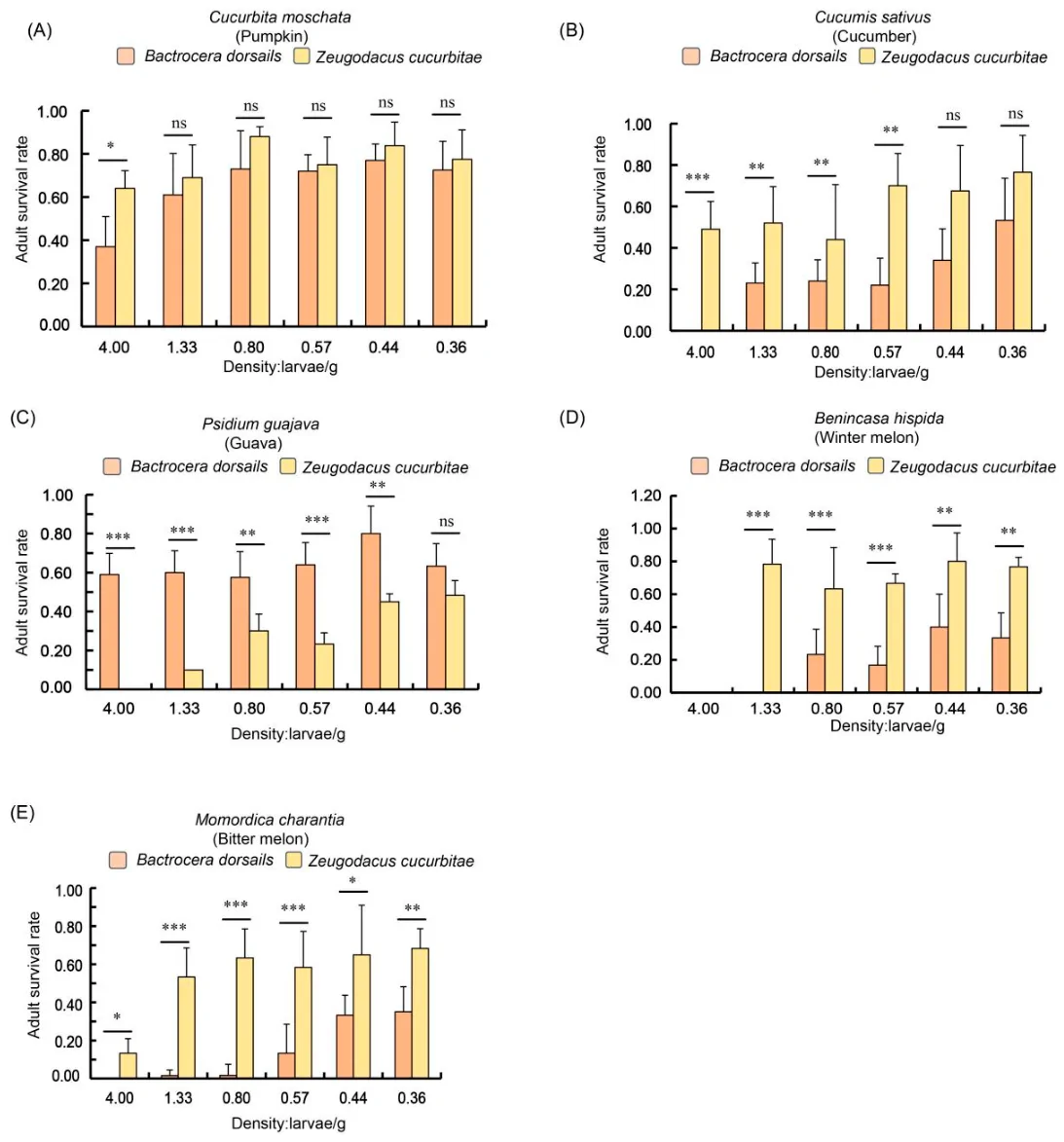
Comparison of adult survival rates between *Z. cucurbitae* and *B. dorsalis* across different host plants and density conditions: (**A**) pumpkin; (**B**) cucumber; (**C**) guava; (**D**) winter melon; and (**E**) bitter melon in single and mixed groups. The standard error is represented by the error bar. ns indicates no significant difference; asterisks indicate significant differences in values determined by multifactorial ANOVA followed by Tukey’s HSD post hoc test (* *p* < 0.05, ** *p* < 0.01, *** *p* < 0.001). For survival rate analysis, data were arcsine-square-root-transformed prior to statistical analysis. “ns” as “no significant”.

The survival rates of both *B. dorsalis* and *Z. cucurbitae* adults exhibited significant variations depending on host plant species and larval density. On pumpkin, the adult survival rate of *Z. cucurbitae* (0.60) was significantly higher than that of *B. dorsalis* (0.35) only under high-density conditions (4 larvae/g), with no significant differences observed at other densities (Figure 1A). The differences were more pronounced on the cucumber host, with values of 0.75 and 0.40, respectively. As the density decreased, the interspecific differences gradually weakened, and most comparisons were not significant at low densities (Figure 1B). In contrast, on guava, *B. dorsalis* exhibited absolute dominance, with significantly higher adult survival rates than *Z. cucurbitae* across both high and low densities (Figure 1C). Conversely, on winter melon and bitter gourd, *Z. cucurbitae* consistently outperformed *B. dorsalis* in survival rates at all tested densities (Figure 1D,E). Notably, when larval density reached its maximum (4 larvae/g), the survival rates of both fruit fly species on winter melon plummeted to near-zero levels, while the survival rate of *B*. *dorsalis* on cucumber and bitter melon was almost completely reduced to zero (Figure 1B,D,E).

### 3.2. Comparison of Adult Dry Weight in Mixed Groups Across Five Host Plants

**Figure 2:**
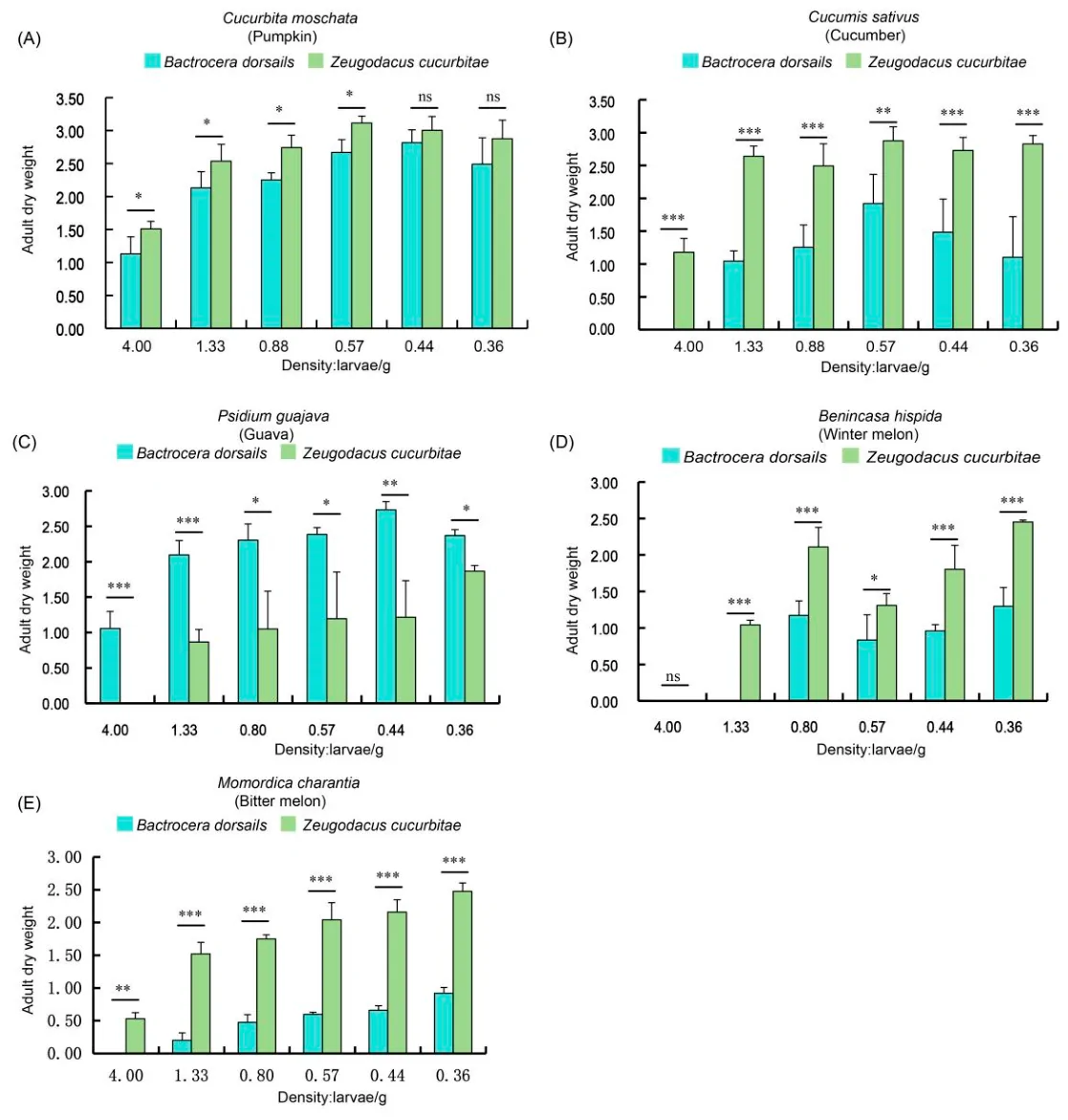
Comparison of adult dry weight of *Z. cucurbitae* and *B. dorsalis* in mixed groups across different host plants and density conditions: (**A**) pumpkin; (**B**) cucumber; (**C**) guava; (**D**) winter melon; and (**E**) bitter melon. The standard error is represented by the error bar. ns indicates no significant difference; asterisks indicate significant differences in values determined by multifactorial ANOVA followed by Tukey’s HSD post hoc test (* *p* < 0.05, ** *p* < 0.01, *** *p* < 0.001). “ns” as “no significant”.

Insect dry weight serves as a critical physiological indicator of organic matter accumulation and can effectively quantify competitive disparities among treatment groups. Our results demonstrate a strong concordance between adult dry weight and survival rates. On four host plants, including pumpkin, cucumber, winter melon, and bitter gourd, *Z*. *cucurbitae* consistently exhibited significantly greater adult dry weight rates than *B*. *dorsalis* across most tested densities (Figure 2A,B,D,E). Notably, on guava, *B. dorsalis* not only achieved a higher dry weight but also demonstrated superior survival rates compared to *Z. cucurbitae* (Figure 2C).

### 3.3. Comparison of Adult Survival Rates Between Single and Mixed Groups of B. dorsalis

**Figure 3:**
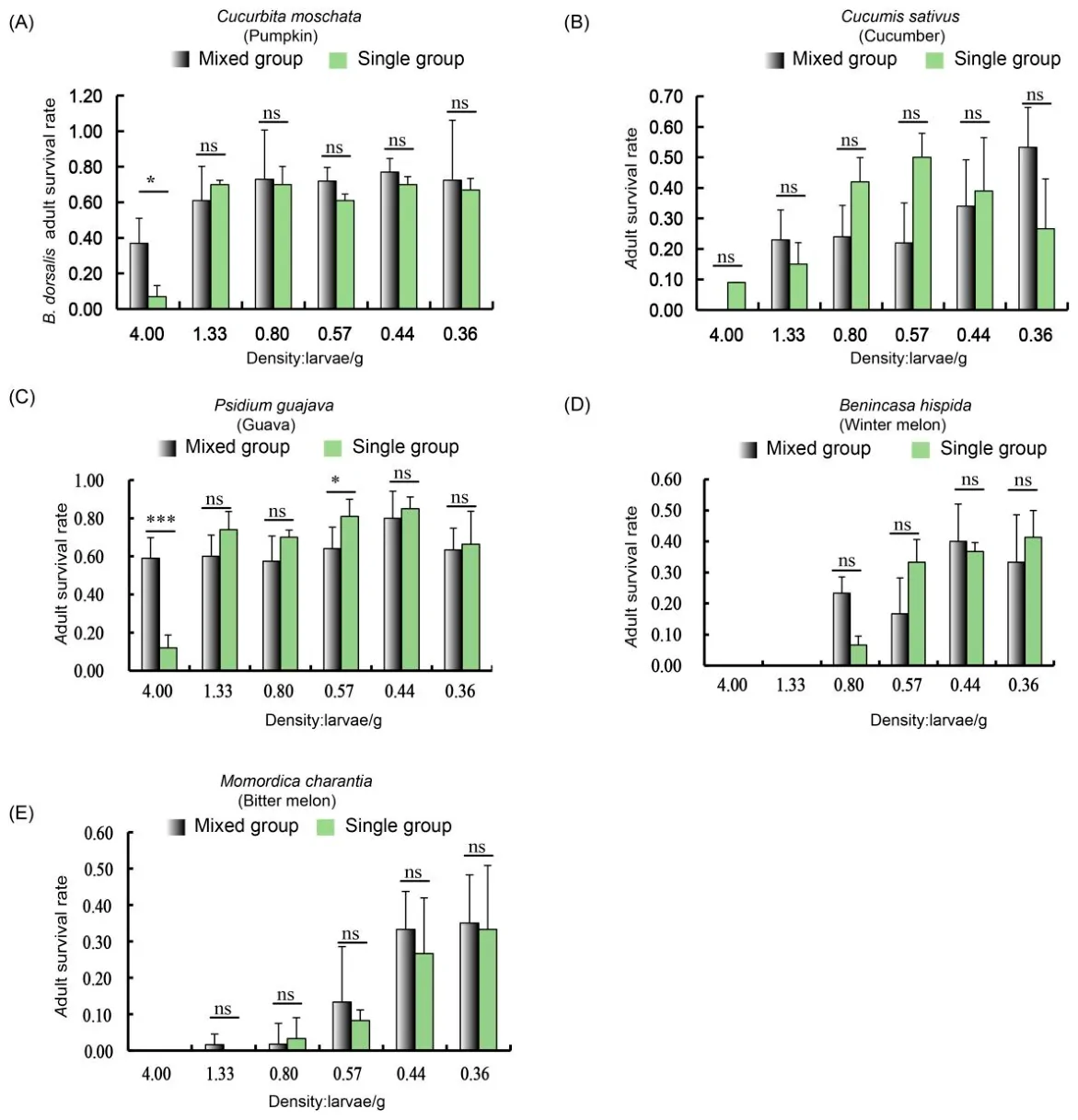
Comparison of adult survival rates of *B. dorsalis* in mixed and single groups across different host plants and density conditions: (**A**) pumpkin; (**B**) cucumber; (**C**) guava; (**D**) winter melon; and (**E**) bitter melon. The standard error is represented by the error bar. ns indicates no significant difference; asterisks indicate significant differences in values determined by multifactorial ANOVA followed by Tukey’s HSD post hoc test (* *p* < 0.05, ** *p* < 0.01, *** *p* < 0.001). For survival rate analysis, data were arcsine-square-root-transformed prior to statistical analysis. “ns” as “no significant”.

To investigate the differences between single and mixed groups of *B*. *dorsalis*, we compared their survival rates at identical larval densities. The results revealed that on pumpkin (Figure 3A), the mixed group exhibited a significantly higher adult survival rate of approximately 0.4 under high-density conditions (4 larvae/g), compared to only 0.1 in the single group. However, no significant differences were observed between the two groups at other density treatments. On cucumber, winter melon, and bitter melon (Figure 3B,D,E), no significant differences in survival rates were detected between solitary and mixed groups across all tested larval densities. Consistent with the pattern observed on pumpkin, on guava (Figure 3C), the mixed group exhibited a competitive advantage only under high-density conditions, while showing no significant difference from the single group at other densities. However, under high-density conditions (4 larvae/g), both single and mixed groups of *B. dorsalis* exhibited extremely low survival rates on winter melon and bitter hosts, approaching zero (Figure 3D,E).

### 3.4. Comparative Analysis of Adult Dry Weight Between Single and Mixed Groups of B. dorsalis

**Figure 4:**
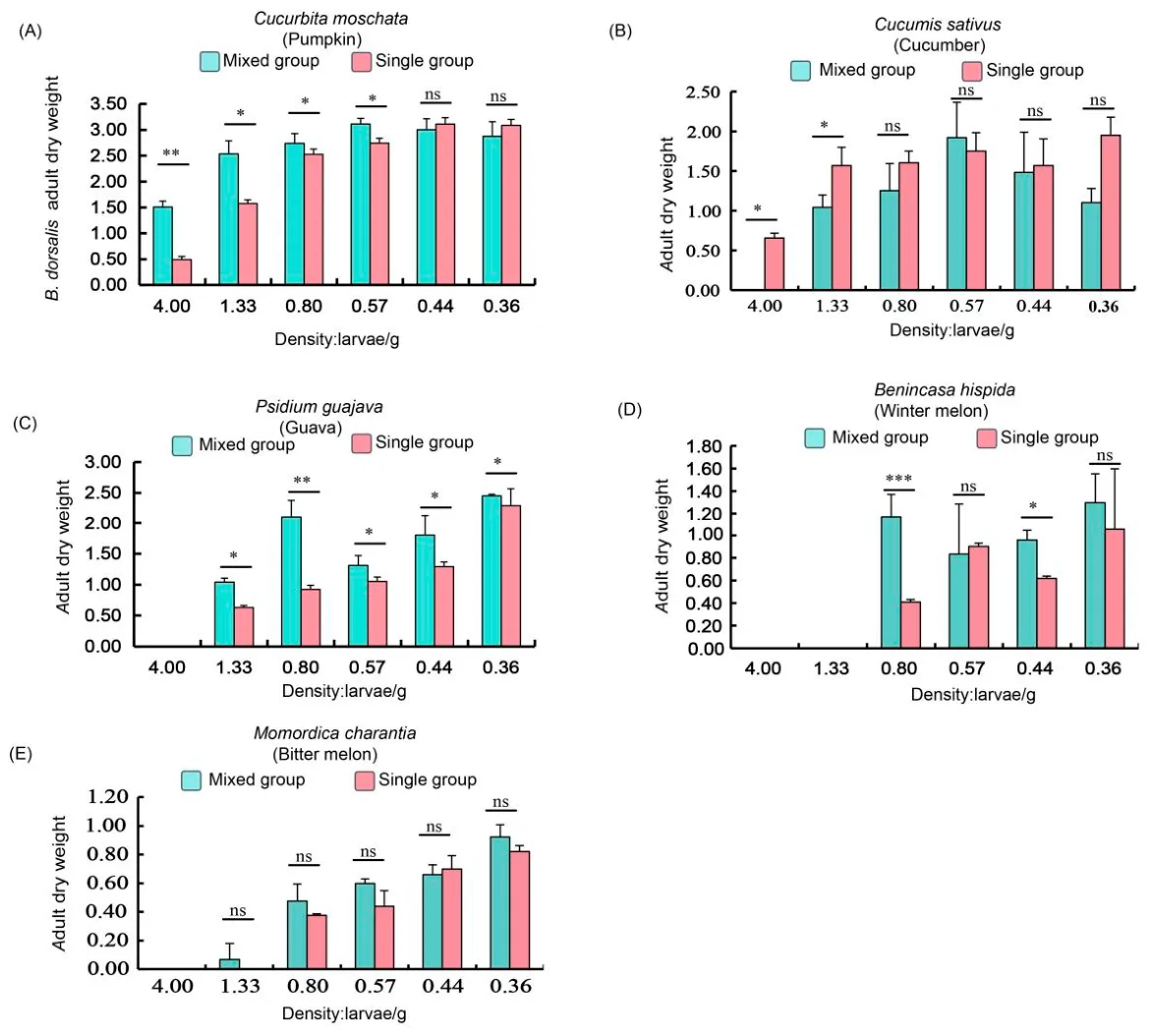
Comparison of adult dry weight of *B. dorsalis* in mixed and single groups across different host plants and density conditions: (**A**) pumpkin; (**B**) cucumber; (**C**) guava; (**D**) winter melon; and (**E**) bitter melon. The standard error is represented by the error bar. ns indicates no significant difference; asterisks indicate significant differences in values determined by multifactorial ANOVA followed by Tukey’s HSD post hoc test (* *p* < 0.05, ** *p* < 0.01, *** *p* < 0.001). “ns” as “no significant”.

The comparative analysis of dry weight revealed distinct host-specific patterns of performance between mixed and single groups: On pumpkin (Figure 4A), the dry weight of adults in the mixed group was significantly higher than that in the single group under high-density conditions (4 and 1.33 larvae/g); in contrast, on cucumber hosts (Figure 4B), the single group exhibited a significantly higher dry weight. Notably, on guava (Figure 4C), the mixed group demonstrated absolute superiority across all tested densities, consistently outperforming the single group in dry weight accumulation. In contrast, on winter melon (Figure 4D), the mixed group only showed a significant advantage over the single group under food-limited conditions (0.8 and 0.44 larvae/g). Remarkably, dry weight did not significantly differ between the two rearing strategies on bitter melon at any tested density (Figure 4E).

### 3.5. Comparison of Adult Survival Rates Between Single and Mixed Groups of Z. cucurbitae

**Figure 5:**
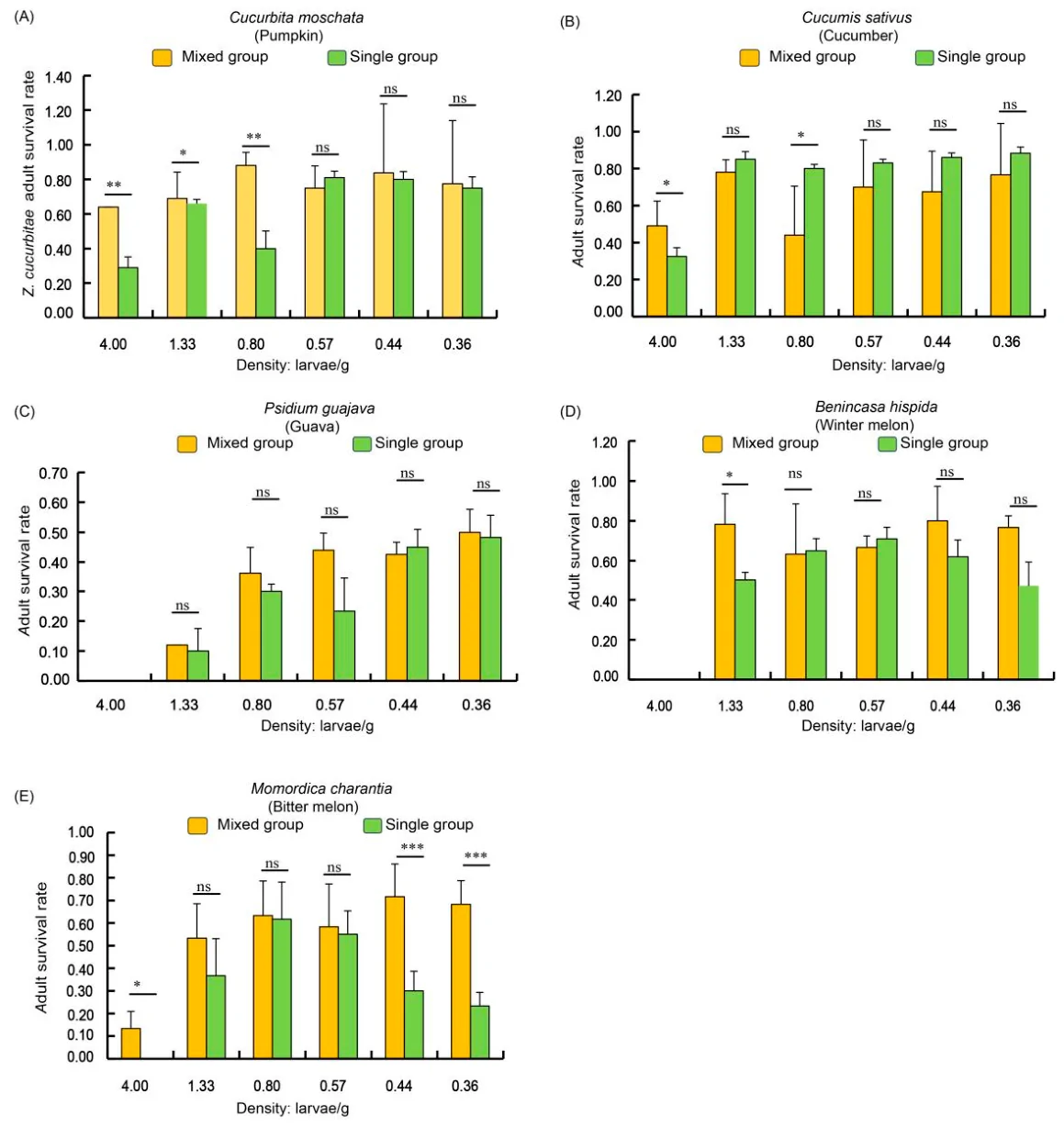
Comparison of adult survival rates of *Z. cucurbitae* in mixed and single groups across different host plants and density conditions: (**A**) pumpkin; (**B**) cucumber; (**C**) guava; (**D**) winter melon; and (**E**) bitter melon. The standard error is represented by the error bar. ns indicates no significant difference; asterisks indicate significant differences in values determined by multifactorial ANOVA followed by Tukey’s HSD post hoc test (* *p* < 0.05, ** *p* < 0.01, *** *p* < 0.001). For survival rate analysis, data were arcsine-square-root-transformed prior to statistical analysis. “ns” as “no significant”.

We investigated the survival rate differences between mixed and single groups of *Z. cucurbitae* under identical larval densities. On pumpkin hosts, the mixed group exhibited significantly higher survival rates than the single group under high-density conditions (4, 1.33, and 0.8 larvae/g), while no significant differences were observed at other densities (Figure 5A). On cucumber, the mixed group showed a significant competitive advantage at the high density of 4 larvae/g, while the single group had an advantage at the medium density of 0.8 larvae/g (Figure 5B). For guava, neither the mixed group nor the single group of *Z. cucurbitae* showed significant differences at any tested density (Figure 5C). Conversely, on winter melon, the mixed group outperformed the single group at low densities (0.36 larvae/g) (Figure 5D). On bitter melon, the mixed group showed a higher survival rate than the single group under both high- and low-density conditions (Figure 5E).

### 3.6. Comparative Analysis of Adult Dry Weight Between Single and Mixed Groups of Z. cucurbitae

**Figure 6:**
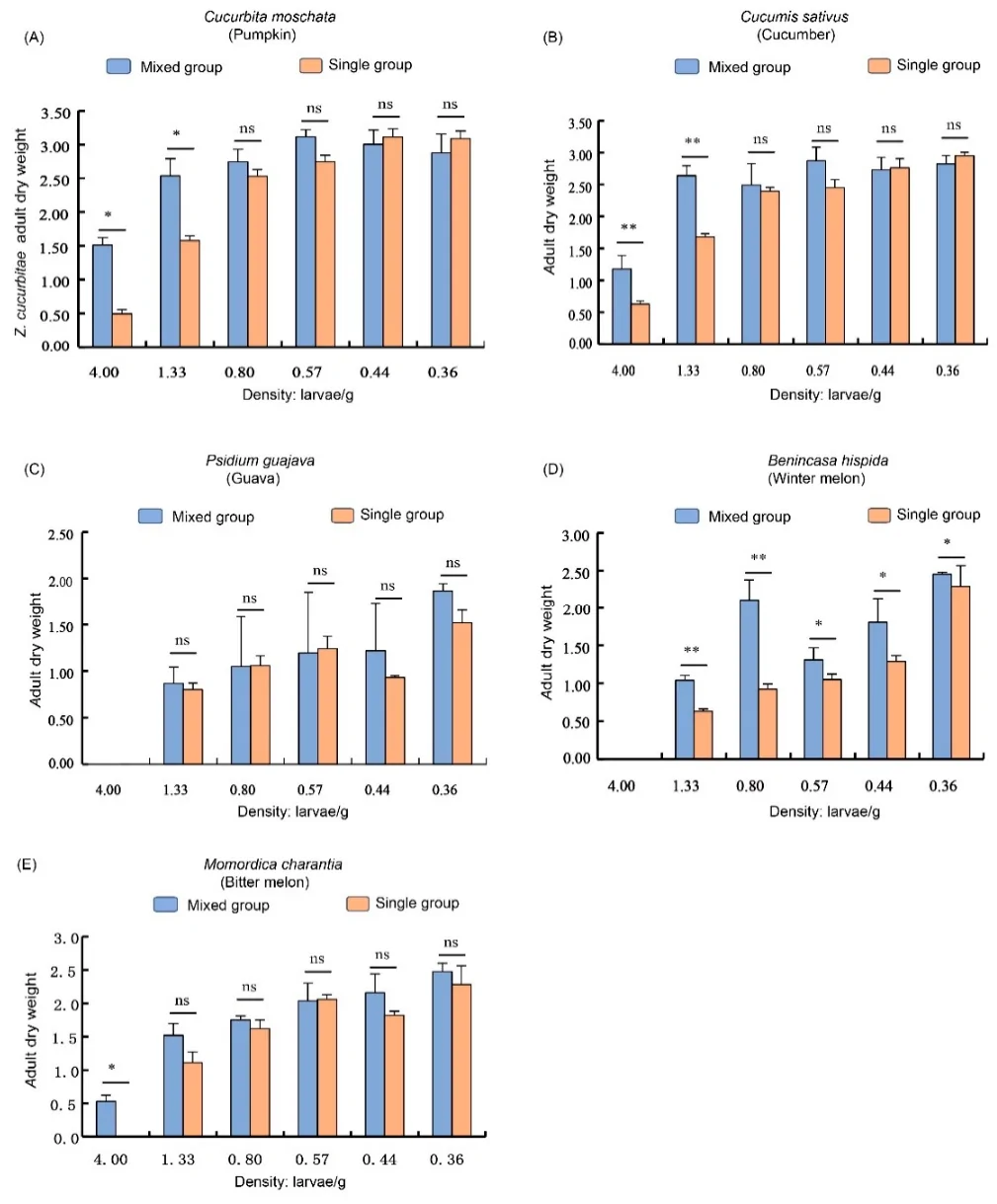
Comparison of dry weight of adult *Z. cucurbitae* in mixed and single groups across different host plants and density conditions: (**A**) pumpkin; (**B**) cucumber; (**C**) guava; (**D**) winter melon; and (**E**) bitter melon. The standard error is represented by the error bar. ns indicates no significant difference; asterisks indicate significant differences in values determined by multifactorial ANOVA followed by Tukey’s HSD post hoc test (* *p* < 0.05, ** *p* < 0.01, *** *p* < 0.001). “ns” as “no significant”.

We investigated the differences in dry weight of larval development into adults between the mixed group and the single group of *Z. cucurbitae* at the same larval density. On pumpkin and cucumber, when the larval density was at high-density conditions (4 and 1.33 larvae/g), the dry weight of adults developed from the mixed group larvae was significantly higher than that of the single group larvae. However, at other density treatments, there were no significant differences in dry weight between the two groups (Figure 6A,B). In the case of guava hosts, there were no significant differences in the dry weight of adults developed from the larvae between the single group and the mixed group under all density conditions (Figure 6C). On winter melon hosts, the dry weight of the mixed group was higher than that of the single group under all density conditions (Figure 6D). In the case of bitter melon hosts, the dry weight of adults developed from the mixed group larvae was only higher than that of the single group larvae when the larval density was at high-density conditions (4 larvae/g) (Figure 6E).

### 3.7. Comprehensive Analysis of Influencing Factors

The multifactorial ANOVA revealed that species, host plant, larval density, and their interactive effects significantly influenced both the dry weight and survival rate of the organisms (Table 1 and Table 2). For dry weight, the independent effects of species, host plant, and larval density all reached highly significant levels (*p* < 0.001): species exhibited a Type III sum of squares of 19.771 (F = 59.938, df = 1, *p* < 0.001), host plant showed a Type III sum of squares of 96.960 (F = 73.565, df = 4, *p* < 0.001), and larval density had a Type III sum of squares of 107.183 (F = 64.989, df = 5, *p* < 0.001). Among these, the host plant demonstrated the most prominent effect size (Type III sum of squares = 96.960), suggesting it is a critical environmental factor influencing dry weight. Furthermore, the species × host plant interaction was highly significant (F = 70.885, df = 4, *p* < 0.001), indicating substantial differences in dry weight across species when reared on different host plants.

For survival rate, species, host plant, and larval density also demonstrated highly significant independent effects (*p* < 0.001). Key interactive effects included the species × host plant interaction, which was highly significant (F = 39.836, df = 4, *p* < 0.001) and played a crucial role in regulating survival rate, as well as the three-way interaction among species, host plant, and larval density, which reached a highly significant level (F = 3.071, df = 8, *p* < 0.001), highlighting the combined effect of these three core factors on organismal survival. Analysis of interactive effects (Table 2) further demonstrated that the species × host plant interaction was a common key determinant for both dry weight (F = 70.885, df = 4, *p* < 0.001) and survival rate (F = 39.836, df = 4, *p* < 0.001), underscoring the pivotal role of host factors in driving intraspecific and interspecific variation in growth and survival. Moreover, the significant three-way interaction among species, host plant, and larval density for survival rate (F = 3.071, df = 20, *p* < 0.001) further revealed the critical regulatory role of multifactorial interactions in organismal adaptation.

## 4. Discussion

Interspecific competition among phytophagous insects can influence survival, development, and population dynamics [22,23]. *Z. cucurbitae* and *B. dorsalis* overlap in host use and oviposition opportunities, providing a basis for potential interspecific interactions on shared fruits [24,25,26,27,28]. The present results indicate that the outcome of these interactions was strongly dependent on host plant, larval density, and species composition, rather than demonstrating a uniform competitive relationship across all tested conditions. Therefore, the observed differences in survival and adult dry weight should be interpreted primarily as density- and host-dependent differences in performance under laboratory conditions, rather than as evidence of universal competitive advantage.

The survival and dry-weight results demonstrated clear differences between the two species across host plants and density treatments. In particular, *Z. cucurbitae* showed higher survival than *B. dorsalis* under the highest tested density (4 larvae g^−1^) on pumpkin, whereas *B. dorsalis* showed higher adult survival and biomass on guava across the tested densities. These results indicate that host identity substantially influenced the relative performance of the two species. The significant effects detected for species, host plant, density, and their interactions further support the importance of these factors in determining the observed variation in survival and dry weight. Thus, rather than assigning a single competitive hierarchy between the two fruit fly species, the results suggest that the relative performance of each species changes according to the host environment and population density.

The density-dependent pattern was particularly evident on cucurbitaceous hosts. When larval density exceeded 1.33 larvae/g, the difference in survival between the two species increased substantially. This pattern is consistent with the expectation that increasing larval density intensifies resource limitation within a standardized substrate. Nevertheless, the present experiments measured adult survival and dry weight and did not directly quantify larval feeding rates, resource depletion, developmental duration, or behavioral interactions among larvae. Consequently, the increased difference in survival at higher densities can reasonably be interpreted as evidence of density-dependent differences in performance, but the underlying mechanism cannot be attributed exclusively to direct interspecific competition. Other density-dependent processes, including resource limitation or differences in tolerance to crowding, may also contribute to the observed pattern.

The dry-weight data provide additional evidence that host and density jointly affected species performance. On pumpkin, mixed-species groups of *Z. cucurbitae* exhibited significantly greater adult dry weight than the corresponding mono-specific groups under the relevant treatment. This result may indicate differences in resource utilization or developmental performance in mixed-species environments. However, greater adult dry weight alone does not demonstrate that *Z. cucurbitae* actively suppressed *B. dorsalis* through competitor interference or superior resource acquisition. Because larval behavior, feeding activity, resource consumption, and individual developmental trajectories were not directly measured, explanations involving competitor suppression or specific physiological mechanisms should therefore be regarded as hypotheses requiring further experimental testing.

Our findings are broadly consistent with previous studies showing that interactions among tephritid fruit flies can vary with host plant and population density [14,18,21,27,29,30,31,32,33,34,35,36]. For example, previous work has reported that differences between competing species become more pronounced at high densities, while outcomes may vary among host fruits [21,27]. Such observations provide useful ecological context for the present results. However, comparisons among studies should be made cautiously because host characteristics, larval densities, experimental substrates, species combinations, and response variables differ among experiments. The present study therefore provides evidence for host- and density-dependent variation in the relative performance of *Z. cucurbitae* and *B. dorsalis* rather than demonstrating that one species is consistently superior across all ecological conditions.

The particularly strong performance of *Z. cucurbitae* on pumpkin at high density may be related to its documented association with cucurbitaceous hosts. Previous studies have reported positive relationships between *Z. cucurbitae* larval survival and host water content and negative relationships with carbohydrate and lipid content [32]. These observations provide a possible ecological explanation for its relatively high performance on cucurbitaceous substrates. However, because the present study did not experimentally manipulate individual nutritional components or measure larval nutrient intake, the contribution of host nutritional composition to the observed differences cannot be established directly. The nutritional theory proposed by Behmer [33] provides a useful conceptual framework, but additional nutritional and physiological experiments are needed to determine whether differences in host nutrient composition explain the patterns observed here.

The results obtained on guava further emphasize the importance of host identity. *B. dorsalis* showed higher adult survival and biomass on guava across the tested densities, contrasting with the pattern observed for *Z. cucurbitae* on pumpkin. This host-dependent reversal indicates that the relative performance of the two species cannot be interpreted independently of host plant identity. The significant species × host interaction observed for survival and dry weight is particularly important in this regard, because it demonstrates statistically that the response of one species relative to the other depended on the host substrate. Such an interaction is more directly supported by the present data than a general claim that either species possesses overall competitive dominance.

The high-density treatments also produced very low or nearly zero emergence of *B. dorsalis* or *Z. cucurbitae* on sponge gourd and bitter gourd; this pattern is compatible with strong density-dependent limitation of successful development under the experimental conditions and may reflect depletion of available resources at high larval densities [37,38]. However, because resource consumption and depletion were not directly quantified, the results do not establish nutrient depletion as the causal mechanism. Future experiments incorporating measurements of substrate nutrient content before and after larval development, larval biomass accumulation, feeding activity, and developmental duration would help distinguish resource limitation from other density-dependent processes.

The present findings also have implications for understanding potential pest-management interactions, but these implications should be interpreted cautiously. The contrasting performance of the two species on cucurbitaceous and non-cucurbitaceous hosts suggests that host-specific monitoring may be useful when both species occur in agricultural landscapes. In particular, the relatively strong performance of *Z. cucurbitae* on pumpkin at high density and the comparatively strong performance of *B. dorsalis* on guava indicate that pest-management priorities may need to consider the dominant host and species present in a production system. However, the current laboratory experiments do not directly demonstrate that suppression of one species will cause population displacement, secondary infestation, or changes in field population abundance. Therefore, recommendations concerning targeted control, interspecific suppression, or shifts between hosts should be considered potential management hypotheses rather than experimentally validated field outcomes [39,40].

The laboratory nature of this study represents an important limitation. Experimental conditions necessarily simplified the ecological environment in which these fruit flies normally develop. In natural fruits, larvae encounter heterogeneous pulp structure, variable moisture distribution, changing nutrient availability, physical barriers, microbial communities, and other environmental conditions that may alter the intensity and outcome of interspecific interactions. In addition, host-fruit availability, fruit size, host diversity, seasonal variation, and larval infestation levels in agricultural fields differ substantially from the standardized and resource-limited substrates used in the present experiments. Natural enemies and other biotic interactions may further modify survival and population dynamics. Consequently, the relative performance patterns observed here should not be directly extrapolated to field-level competitive exclusion or population regulation without field validation.

Importantly, the present study measured adult survival and dry weight as principal indicators of performance. These variables provide useful evidence for differences in successful development and adult condition, but they do not directly reveal the behavioral, physiological, nutritional, or metabolic mechanisms responsible for those differences. Accordingly, interpretations concerning larval feeding strategies, physiological adaptation, resource acquisition, or mechanisms of competitive suppression should remain cautious. Future research should integrate behavioral observations, larval developmental measurements, resource-consumption assays, nutritional profiling, and physiological or molecular analyses to determine the mechanisms underlying the host- and density-dependent patterns observed here.

Overall, the results support a context-dependent interpretation of interactions between *Z. cucurbitae* and *B. dorsalis*. The significant species × host and species × host × density effects indicate that the relative performance of the two species changed according to the combination of host identity and larval density. The strongest evidence for differential performance occurred on pumpkin at high density, where *Z. cucurbitae* showed higher survival and/or dry weight, whereas *B. dorsalis* performed better on guava. These findings suggest that host characteristics and population density are important determinants of the outcome of interactions between these economically important fruit flies. However, whether these laboratory patterns represent direct interspecific competition, differential resource exploitation, crowding tolerance, or other ecological processes remains to be determined experimentally.

## 5. Conclusions

This study demonstrates that the relative performance of *Z. cucurbitae* and *B. dorsalis* varies substantially with host plant and larval density under controlled laboratory conditions. The results show that species, host, density, and their interactions significantly influence adult survival and dry weight, with *Z. cucurbitae* performing particularly well on pumpkin under high-density conditions, whereas *B. dorsalis* showed comparatively greater performance on guava. These findings indicate that interactions between the two fruit fly species are context-dependent rather than characterized by a single consistent competitive hierarchy.

The study provides a useful experimental basis for understanding how host identity and population density may influence the performance of co-occurring tephritid species. From an applied perspective, the findings support the value of host-specific monitoring and management strategies; however, direct field-level recommendations concerning competitive displacement or population suppression require further validation. Future studies should examine these interactions under natural fruit conditions and field environments while incorporating broader host diversity, realistic density gradients, natural enemies, and seasonal environmental variation. Integrating behavioral, nutritional, physiological, and molecular measurements, together with more appropriate binomial statistical models such as GLM/GLMM approaches with a logit link, will also help clarify the mechanisms responsible for the observed patterns.

Therefore, the principal contribution of this study is not evidence of universal competitive dominance by either species, but rather the demonstration that host identity and larval density jointly shape the relative performance of *Z. cucurbitae* and *B. dorsalis*. This context-dependent perspective provides a stronger foundation for future investigations of tephritid interactions and for developing evidence-based pest-management strategies.

## Figures and Tables

**Table 1:** The environmental factors and levels.

Species	Host Plant	Density (Larvae/g)	Group Type
*Zeugodacus cucurbitae*	*Cucurbita moschata*	4.0	Single group
*Bactrocera dorsalis*	*Cucumis sativus*	1.33	Mixed group
	*Benincasa hispida*	0.8	
	*Momordica charantia*	0.57	
	*Psidium guajava*	0.44	
		0.36	

**Table 2:** The multi-factor ANOVA on the effects of environmental factors on the survival rate and dry weight of the melon fruit fly (*Zeugodacus cucurbitae*) and the oriental fruit fly (*Bactrocera dorsalis*).

Response Variable	Factor/Interaction	Type III Sum of Squares	df	F Value	*p* Value	Significance
Survival rate	Species	3.986	1	60.158	0.000	***
	Host plant	10.558	4	39.836	0.000	***
	Density	15.904	5	48.006	0.000	***
	Group type	0.256	1	3.867	0.050	*
	Species × Host plant	18.787	4	70.885	0.000	***
	Species × Density	0.625	5	1.886	0.096	ns
	Species × Group type	0.045	1	0.683	0.409	ns
	Host plant × Density	2.608	20	1.968	0.008	**
	Host plant × Group type	1.881	4	7.097	0.000	***
	Density × Group type	0.890	5	2.686	0.021	*
	Species × Host × Density	4.829	20	3.644	0.000	***
	Species × Host plant × Group type	0.152	4	0.574	0.681	ns
	Species × Density × Group type	0.115	5	0.348	0.883	ns
	Host plant × Density × Group type	2.019	20	1.524	0.069	ns
	Species × Host plant × Density × Group type	2.318	20	1.749	0.024	*
Dry weight	Species	19.771	1	59.938	0.000	***
	Host plant	96.960	4	73.488	0.000	***
	Density	107.183	5	64.989	0.000	***
	Group type	4.659	1	14.125	0.000	***
	Species × Host plant	97.062	4	73.565	0.000	***
	Species × Density	5.004	5	3.034	0.011	*
	Species × Group type	0.545	1	1.652	0.199	ns
	Host plant × Density	21.782	20	3.302	0.000	***
	Host plant × Group type	4.034	4	3.057	0.017	*
	Density × Group type	6.385	5	3.871	0.002	**
	Species × Host plant × Density	15.930	20	2.415	0.001	***
	Species × Host plant × Group type	5.300	4	4.017	0.003	**
	Species × Density × Group type	0.503	5	0.305	0.910	ns
	Host plant × Density × Group type	12.223	20	1.853	0.014	ns
	Species × Host × Density × Group type	5.287	20	0.801	0.713	ns

* *p* < 0.05, ** *p* < 0.01, *** *p* < 0.001, “ns” as “no significant”.

## Data Availability

The data presented in this study are available upon request from the corresponding authors.
